# Repopulation of a 3D simulated periapical lesion cavity with dental pulp stem cell spheroids with triggered osteoblastic differentiation

**DOI:** 10.1590/0103-644020235847

**Published:** 2024-12-16

**Authors:** Vítor Luís Ribeiro, Janaína A. Dernowsek, Roger R. Fernandes, Dimitrius L. Pitol, João Paulo Mardegan Issa, Jardel F. Mazzi-Chaves, Karina Fittipaldi Bombonato-Prado, Manoel Damião Sousa-Neto, Geraldo Aleixo Passos

**Affiliations:** 1 Program in Restorative Dentistry, Department of Restorative Dentistry, School of Dentistry of Ribeirão Preto, University of São Paulo(USP), Ribeirão Preto, SP, Brazil.; 2 Biotechnology Center, Energy and Nuclear Research Institute (IPEN), University of São Paulo, São Paulo, SP, Brazil; 3 Department of Bucomaxilofacial Surgery, School of Dentistry of Ribeirão Preto, University of São Paulo (USP), Ribeirão Preto, SP, Brazil.; 4 Department of Basic and Oral Biology, School of Dentistry of Ribeirão Preto, University of São Paulo(USP), Ribeirão Preto, SP, Brazil.; 5 Center for Cell-Based Therapy in Dentistry, School of Dentistry of Ribeirão Preto, University of São Paulo(USP), Ribeirão Preto, SP, Brazil.; 6 Laboratory of Genetics and Molecular Biology, Department of Basic and Oral Biology, School of Dentistry of Ribeirão Preto, University of São Paulo(USP), Ribeirão Preto, SP, Brazil.; 7 Molecular Immunogenetics Group, Department of Genetics, Ribeirão Preto Medical School, USP, Ribeirão Preto, SP, Brazil.

**Keywords:** periapical lesion, stem cells, spheroids, 3D printing, regenerative endodontics

## Abstract

We established a proof-of-concept model system for the biological healing of periapical lesions using stem cell spheroids. Mesenchymal stem cells from human exfoliated deciduous teeth (SHED) were cultured in a 2D monolayer and then as 3D multicellular spheroids. An image of a periapical lesion of an upper lateral incisor tooth was obtained by computed tomography and was used as a model for photopolymer resin 3D printing to generate a negative frame of the lesion. The negative model served to prepare a positive model of the periapical lesion cavity in an agarose gel. SHED that were cultured in monolayers or as spheroids were seeded in the positive lesion mold before or after osteoblastic differentiation. The results showed that compared to cells cultured in monolayers, spheroids exhibited uniform cellularity and a greater viability within the lesion cavity, which was accompanied by a temporal reduction in the expression of CD13, CD29, CD44, CD73, and CD90 mRNAs that are typically expressed by stem cells. Concomitantly, the expression of markers that characterize osteoblastic differentiation (RUNX2, ALP, and BGLAP) increased. These results provide a new perspective for regenerative endodontics with the use of SHED-derived spheroids to repair periapical lesions.

## Resumo

Estabelecemos um modelo de prova de conceito para o tratamento biológico de lesões periapicais utilizando esferoides de células-tronco. Uma linhagem de células- tronco mesenquimais isoladas da polpa dentária de dentes decíduos (células SHED) foi cultivada em monocamada 2D e posteriormente em esferoides multicelulares em 3D. Uma imagem de uma lesão periapical de um incisivo lateral superior foi obtida por tomografia micro computadorizada, a qual foi usada como modelo para impressão 3D de resina fotopolimérica, gerando um molde negativo da lesão. O modelo negativo foi utilizado para preparar um modelo positivo da cavidade da lesão periapical em gel de agarose. Células SHED cultivadas em monocamadas ou como esferoides foram semeadas no molde positivo da lesão antes ou após a diferenciação osteoblástica. Os resultados mostraram que, em comparação com células cultivadas em monocamadas, os esferoides apresentaram celularidade uniforme e maior viabilidade dentro da cavidade da lesão, acompanhada de redução temporal na expressão de RNAs mensageiros tipicamente expressos por células-tronco (CD13, CD29, CD44, CD73 e CD90). Concomitantemente, houve um aumento na expressão de marcadores proteicos que caracterizam a diferenciação osteoblástica (RUNX2, ALP e BGLAP). Esses resultados proporcionam uma nova perspectiva para a endodontia regenerativa com o uso de esferoides preparados com células SHED para reparar lesões periapicais.

## Introduction

Anaerobic multi-bacterial infection of the dental pulp and root canals causes inflammation and periapical injury. Inflammation may damage periapical tissues, resulting in the reabsorption of the bone around the root apex ^1^. For the periapical lesion to heal, it is necessary to eliminate or reduce bacterial contamination in the root canal. These goals are achieved through endodontic treatment, which initially consists of biomechanical preparation of the root canal system using irrigating solutions to remove necrotic organic tissues, intracanal medications, and canal filling, followed by restorative processes. This treatment allows for the restoration of dental element function ^2^ ^,^ ^3^, and subsequent sealing prevents microleakage or new bacterial contamination of the root canal system.

Periapical injury is a multifactorial disease, with factors other than endodontic and restorative treatments involved. These factors, such as bacterial microbiology related to the intrinsic characteristics of the host, including the ability to defend themselves in response to the contaminating agent or to heal after contaminant removal, must be considered. Thus, the relationship between the host resistance and microbial virulence directly influences the development and healing of disease. In other words, not all individuals have the same physiological response to endodontic treatment, which can be explained by individual genetic backgrounds ^4^ ^,^ ^5^.

There is a research gap regarding the success of endodontic treatment in patients who do not show regeneration of periapical lesions, even after endodontic treatment or retreatment ^6^ ^,^ ^7^. These patients could benefit from therapies that use stem cells. This approach aims to regenerate persistent or critically sized periapical lesions and shows promise in curing the disease ^8^ ^,^ ^9^.

Stem cells from human exfoliated deciduous teeth (SHED) are mesenchymal stem cells obtained from dental pulp explants ^10^. SHED are widely utilized in studies because of their high proliferation and differentiation rates. These cells are characterized by mesenchymal stem cell markers, such as OCT4, CD13, CD29, CD44, CD73, CD90, CD105, CD146, and CD166 ^11^ ^,^ ^12^. SHED have been shown to be important in the regeneration and repair of craniofacial injuries, tooth loss, and bone loss, owing to their high proliferation and differentiation capacities; they may be suitable for regenerative endodontic therapy of critically sized bone lesions and persistent periapical lesions ^8^ ^,^ ^12^.

Monolayer (2D) mammalian adherent cell culture has been the gold standard method for cell biology studies for many years. However, changes in cell properties may occur, leading to differences relative to the *in vivo* microenvironment in the organism ^13^ ^,^ ^14^. Mammalian cells, in general, are very responsive. Depending on the physical or chemical stimulus, they can show adaptive changes in their biochemical or physiological mechanisms. To reduce the effects of monolayer (2D) culture and more closely mimic *in vivo* conditions, three-dimensional (3D) cell cultures have been developed ^15^. A 3D cellular structure can be achieved via the formation of nonadherent multicellular spheroids, which show a more remarkable similarity of their biochemical and physiological responses to those of *in vivo* tissues than do adherent cells cultured in 2D monolayers ^16^.

Considering the high capacity of stem cells from human exfoliated deciduous teeth (SHED) for *in vitro* osteoblastic differentiation ^17^, we hypothesized that these cells might grow and differentiate in a simulated periapical lesion cavity.

Therefore, the aim of this work was to establish an *in vitro* experimental model system for biological repopulation of a simulated periapical lesion cavity using SHED. For this purpose, we used SHED to form 3D spheroids *in vitro.* The need for this model is related to *in vitro* mimicking an actual periapical lesion to test the concept of repopulation using stem cells.

We compared the osteoblastic differentiation capacity of spheroids to that of SHED cultured in 2D monolayers. Osteoblasts represent a differentiation stage immediately preceding mineralization and calcium deposition, which are crucial for healing the lesion cavity. Additionally, we compared spheroids and cells cultured in 2D monolayers after osteoblastic differentiation within an actual 3D simulated periapical lesion model.

## Materials and Methods

### Mesenchymal stem cells from human exfoliated deciduous teeth (SHED)

The SHED utilized in this study were previously isolated ^18^. The cells were obtained from a healthy male child, cultured in DMEM supplemented with 15% fetal bovine serum (FBS) and 100 U/ml penicillin and streptomycin (hereafter referred to as control medium or CMD medium) and incubated at 37 °C in an atmosphere of 5% CO2. These cells were previously assayed by flow cytometry using a BD FACS Calibur flow cytometer (Becton Dickinson and Company, Franklin Lakes, NJ) to confirm the expression of the surface markers CD29, CD44, CD13, CD90, and CD73 ^12^. This cell line showed no contamination with hematopoietic stem cells, as determined by the assessment of the surface markers CD34 and CD45/CD14 ^12^.

In this study, cells at passage four were cultured as both conventional adherent 2D monolayers and nonadherent 3D spheroids in Dulbecco’s modified Eagle’s medium (DMEM) supplemented with nutrient mixture F-12 (DMEM/F12; Gibco®) and 15% inactivated fetal bovine serum in an incubator at 37 °C with a 5% CO2 atmosphere.

SHED samples cultured in DMEM/F12 medium supplemented with chemical osteoblastic inducers, as specified below, were collected at three time points during differentiation, namely, after 3, 7, and 10 days. Cell viability was assessed via the CyQuant^TM^ MTT cell viability assay (Thermo Fisher), which relies on the metabolism of MTT by viable cell mitochondria, with colorimetric absorbance readings at 570 nm. The colorimetric absorbance readings were made using a Synergy Biotek microplate reader.

All the procedures involving the sampling of human cells were approved by the Committee for Ethics in Research of the Biosciences Institute, USP, São Paulo, Brazil (approval #435054), for Dr. Maria Rita S. Passos-Bueno who isolated the cells and established the cell line. She kindly supplied the SHED from the original stock for this study.

### Osteoblastic differentiation of SHED in vitro

The chemical induction of the differentiation of SHED cultured in 2D monolayers into osteoblasts was triggered by the addition of 10^-7^ M dexamethasone (Sigma), 5 µg/mL ascorbic acid (Gibco), and 2.16 g/mL β-glycerophosphate (Sigma) to DMEM/F12 culture medium supplemented with 15% FBS ^12^. During the entire culture time, the cells were kept at 37 °C in an atmosphere with 5% CO2. The culture medium containing the chemical inducers was changed every three days.

Samples were collected at three time points during differentiation, i.e., after 7, 14, and 21 days, for cell viability and osteoblastic differentiation assays, such as the Fast Red and mineralization assays, immunofluorescence staining with antibodies recognizing osteoblastic cell markers and RT‒qPCR to quantify the expression of osteoblast-related mRNAs.

Moreover, at 7 and 14 days of exposure to the inducing agents, the cells were removed from the culture flasks by trypsinization and seeded in 600-µm or periapical lesion agarose micromolds to obtain spheroids during the osteoblastic differentiation process.

The monitoring of osteoblastic differentiation was performed using the methods described below.

### Detection of alkaline phosphatase (ALP)

Briefly, SHED was cultured in the presence or absence of inducing agents in 24- well plates and were evaluated at 7, 14, and 21 days. The cells were observed to be at 80%, 100% and 100% confluence, respectively. ALP activity was detected by the reaction with Fast Red (Sigma-Aldrich). After staining, the liquid medium was removed, and the plates were allowed to dry for 48 hours at room temperature and photographed. The images were analyzed with the ImageJ program available at https://imagej.nih.gov/ij/download.html.

### Mineralization

Mineralization (calcium deposition areas) was evaluated by staining cells with alizarin red ^12^. SHED was grown in the presence or absence of inducing agents in 24- well plates and assessed at 7, 14 and 21 days; the cells were observed to be at 95%, 100% and 100% confluence, respectively. The liquid medium was removed from the cultures, and the wells were washed three times with PBS at 37 °C, then dehydrated with 70% ETOH for one hour at 4 °C, washed with PBS and stained with Alizarin Red (Sigma- Aldrich) for 45 minutes. Afterward, the wells were washed with PBS and deionized water and allowed to dry for 48 hours at room temperature. The wells were photographed, and the images were analyzed using the ImageJ program.

### Immunofluorescence staining

For immunofluorescence staining, we used a previously described protocol ^19^. Briefly, SHED grown in a monolayer on glass coverslips in control medium or a medium supplemented with chemical inducers for 7, 14, or 21 days were fixed with 4% paraformaldehyde in PBS (pH 7.2) for 10 minutes at room temperature.

Cell permeabilization was performed with a 0.5% Triton X-100 solution in PBS for 10 minutes, followed by blocking with a 5% skim milk solution in PBS for 30 minutes. Primary monoclonal antibodies against RUNX2 (IgG mouse anti-human monoclonal antibody from Abcam, UK; 1:100 dilution), ALP (IgG1 mouse anti-human B4-78 monoclonal antibody from Developmental Studies Hybridoma Bank, USA; 1:100 dilution), or BSP (IgG2a mouse anti-rat WV1D1 (9c5) monoclonal antibody from Developmental Studies Hybridoma Bank, USA; 1:200 dilution) were incubated with the cells for 1 hour.

After incubation with primary antibodies, the cells were stained with a goat anti- mouse secondary antibody conjugated with Alexa Fluor 594 (Molecular Probes, Thermo Fisher Scientific, USA) at a 1:200 dilution for 50 minutes. For the observation of cell limits and cell nuclei, phalloidin conjugated with Alexa Fluor 488 (Molecular Probes, USA) at a 1:200 dilution was used, and the cells were incubated for 50 minutes with 4´,6´- diamidino-2-phenylindole (DAPI) (Molecular Probes, Thermo Fisher Scientific, USA) for 5 minutes. Antifade Prolong mounting medium (Molecular Probes, USA) was used, and labeling was analyzed using a Leica fluorescence microscope model DMLB (Leica Microsystems, Wetzlar, Germany).

### Formation of spheroids from 2D SHED monolayers

**Supplemental Figure 1:**
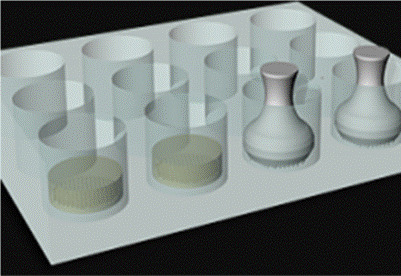
- Manufacturing of 600 µm positive micro-molds in an agarose gel.

In this study, microwells in a 2% agarose gel prepared with sterile PBS were used and molded on a set of 600-µm photopolymer resin pins (Supp. Figure 1).

The agarose microwells were filled with a SHED suspension (1.25 x 10^5^ cells/ml in DMEM/F12 medium) prepared from conventional 2D monolayer cultures. The agarose microwells with the SHED were incubated in 12-well plates at 37 °C and 5% CO2 for 24 to 96 hours to allow the formation of multicellular spheroids, whose formation was observed by using a Cytosmart^TM^ live cell imaging system (Lonza).

### Simulation of a periapical lesion cavity by 3D printing

An *in vitro* model system to mimic periapical lesions was used in this study. It was made by scanning the area of a periapical lesion of a tooth that needed endodontic treatment. This area was obtained using cone-beam computed tomography of an upper lateral incisor tooth of a patient from the collection of tomographic examinations at the Endodontics Clinic at the Ribeirão Preto School of Dentistry, University of São Paulo, Ribeirão Preto, SP, Brazil. This study aimed to precisely replicate the anatomical conditions observed in patients rather than using a randomly selected three-dimensional mold. Our study highlights the importance of using a replica of the periapical lesion cavity to accurately assess cellular behavior in this context.

Segmentation of the file was conducted using the CTAn program v.1.18.4.0+ (SkyScan, Kontich, Belgium) with the aid of a contrast histogram. This involved discerning differences in the gray density to segment and separate the structure comprising the periapical lesion from adjacent structures, such as the tooth and alveolar bone.

The 3D periapical lesion molds were crafted through reverse engineering using the Rhinoceros® 5.0 software (McNeel North America, Seattle, WA, USA), which generated a stereolithographic file (STL) reflecting the scanned periapical lesion. This method ensured the creation of a periapical lesion model with accurate dimensions.

**Supplemental Figure 2:**
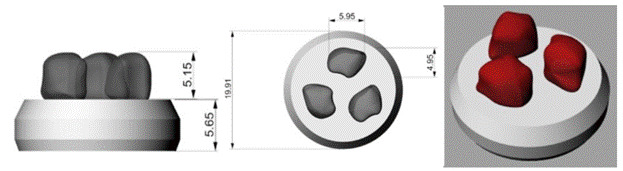
- Negative mold made by 3D printing that reproduces a periapical lesion of an upper lateral incisor tooth.

The molds were fabricated using 3D printing technology. The STL file was processed using digital light processing (DLP) on a SparkMaker® 3D printer (LCD-E, SparkMaker Corporation, Shenzhen, China) in resin to produce negative molds mirroring the exact shape and measurements of the scanned periapical lesion. Each 3D mold was designed for placement in 12-well culture plates, accommodating three periapical lesions of identical shapes and measurements per mold (Supp. Figure 2). Positive molds on agarose were created following the agarose gel preparation protocol outlined earlier.

These positive molds replicated the actual periapical lesion, serving as the substrate for subsequent seeding of SHED under two different conditions (groups): 1)2D-PL, utilizing SHED from monolayer cultures post osteoblastic differentiation, and 2) 3D-PL, employing multicellular 3D SHED spheroids from 600-µm pin micromolds after osteoblastic differentiation.

### Construction of growth curves of spheroids

Two spheroid growth curves were constructed, one for each type of agarose micromold, i.e., 600-µm micromolds and micromolds mimicking the periapical lesion. For the growth curve in 600-µm microwells, 1.25 x 10^5^ cells were seeded, and the spheroids were dispersed by conventional trypsin treatment 24, 48, 72, and 96 hours after SHED seeding. For the growth curve of spheroids growing in the micromolds of the periapical lesion, 3.0 x 10^5^ cells were seeded, and the spheroids were dispersed with trypsin 8, 16, 24, 32, and 40 hours after seeding. The cells were stained with trypan blue and counted with an automatic cell counter (Nexcelom Auto T4 Cellometer). For each curve, the arithmetic mean from three determinations for each time point and the standard deviation of the mean were calculated.

### Spheroid histology

The spheroids formed in the 600-µm micromolds or the periapical lesion micromolds were harvested during the exponential growth phase. The spheroids were fixed with 10% buffered formaldehyde (pH 7.0) and dehydrated in ethanol using a conventional hematoxylin-eosin (H&E) histological protocol. The dehydrated material was mounted on a microscopic slide, stained with H&E, and covered with a coverslip for later examination under an optical microscope (Leica, Germany).

### Spheroid viability

A qualitative assessment of the viability of spheroids formed in 600-µm micromolds or in micromolds of the periapical lesion at the exponential growth phase was performed using the Live/Dead^TM^ viability/cytotoxicity kit (Invitrogen-Thermo Fisher Scientific) according to the manufacturer's instructions. The procedure discriminates between live and dead cells based on staining with green fluorescent calcein-AM as an indicator of the intracellular esterase activity in live cells and red fluorescent ethidium homodimer-1 as an indicator of the loss of plasma membrane integrity by dead cells.

Images were recorded using a fluorescence microscope (Nikon model Ti 5). The regions of spheroids stained green represented living cells, and those stained red represented dead cells.

### Reverse transcription-quantitative real-time PCR (RT‒qPCR)

RT‒qPCR was used to evaluate the transcription levels of mRNAs involved in osteoblastic differentiation or those related to the stemness of mesenchymal stem cells. The mRNAs (cDNAs) are indicated as follows, with the respective GenBank accession numbers and the sequences of the respective 5′ to 3′ forward and reverse oligonucleotide primers in parentheses:

GAPDH acc, (ACGACCAAATCCGTTGACTC and CTCTGCTCCTCCTGTTCGAC);

RUNX2 acc, (AGTAAGAAGAGCCAGGCAGG and GCTGGATAGTGCATTCGTGG);

ALP acc, (CCACGTCTTCACATTTGGTG and AGACTGCGCCTGGTAGTTGT);

BGLAP acc, (GGCAGCGAGGTAGTGAAGAG and CTGGAGAGGAGCAGAACTGG);

SP7 acc, (TGCTTGAGGAGGAAGTTCAC and AGGTCACTGCCCACAGAGTA);

BSP acc, (ACAACACTGGGCTATGGAGA and CCTTGTTCGTTTTCATCCAC);

CD13 acc, (GCCGTGTGCACAATCATCGC and CACCAGGGAGCCCTTGAGGT);

CD29 acc, (ACCAAGGTAGAAAGTCGGGA and TGACCACAGTTGTTACGGCA);

CD44 acc, (ACTGCAATGCAAACTGCAAG and AAGGTGGAGCAAACACAACC);

CD73 acc, (GTTCTCCCAGGTAATTGTGC and ACCTGAGACACACGGATGAA);

CD90 acc, (GCCCTCACACTTGACCAGTT and GCCTTCACTAGCAAGGACGA).

Thermal cycling was completed using a StepOne real‐time PCR system (Applied Biosystems) as follows: 50 °C for 2 min, 95 °C for 15 min, and 60 °C for 1 min (40 cycles). The 2^−ΔΔCT^ method was used for relative quantification. The GAPDH mRNA expression level was used as an internal housekeeping normalizer. We used the GraphPad Prism 5.00 tool (http://www.graphpad.com/prism/Prism.html) to perform one‐way ANOVA with Bonferroni correction to compare the expression levels of mesenchymal stemness-related mRNAs (CD13, CD29, CD44, CD73 and CD90) between noninduced and induced SHED at 7 and 14 days. To compare the expression levels of osteoblastic differentiation-related mRNAs (RUNX2, ALPL, BGLAP, SP7 and BSP) in induced cells at 7 and 14 days, an unpaired t test was used. For both types of statistical tests, p ˂ 0.05 was considered significant.

## Results

### Osteoblastic differentiation of SHED in monolayers and spheroids

Owing to the importance of alkaline phosphatase (ALP) in the mineralization and bone formation processes, ALP protein expression was measured using the Fast Red technique. The measurements (n = 5) were taken at 7, 14 and 21 days of differentiation of SHED in a monolayer culture in the presence of osteoblastic differentiation-inducing agents.

**Figure 1:**
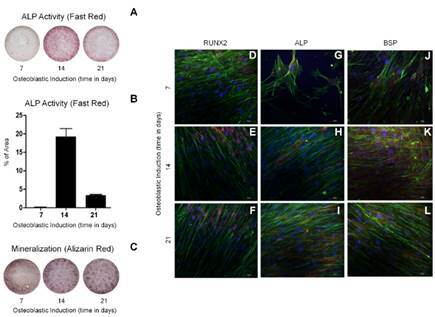
Osteoblastic differentiation of SHED cells in monolayers. SHED cells were cultured in the presence of osteoblastic differentiation medium. The fast-red technique quantified the activity of alkaline phosphatase (ALP) in 7, 14 and 21 days. **(A, B)** The ALP activity peak occurred at 14 days, with the most significant decline at 21 days in culture. Mineralization was also evaluated during differentiation by staining with alizarin red. **(C)** The highest concentration of mineralization nodules occurred at 21 days in culture. **(D-L)** Immunolocalization of differentiation markers RUNX2, ALP, and BSP was also used. SHED was cultured in the presence of an induction medium and observed after 7, 14 and 21 days. It was possible to follow from the 14 days in culture that the SHED exhibited the three marker proteins.

We observed an increase in the ALP activity on days 7 and 14 and a decrease on day 21 during *in vitro* osteoblastic differentiation of SHED (Figure 1A-B). Stained SHED (n = 5) at 7, 14 and 21 days of osteoblastic differentiation were imaged. Imaging analysis revealed the presence of darkened areas as an indicator of mineralized nodules between 17 and 21 days (Figure 1C).

Immunolocalization of three osteoblastic differentiation markers, namely, the transcription factor RUNX2, the ALP enzyme, and bone sialoprotein (BSP), was performed after 7, 14 and 21 days of SHED differentiation in monolayer cultures. RUNX2 and ALP were expressed during the three analyzed periods, and RUNX2 was located in the nucleus (Figure 1D-F), ALP was located on the cell surface (Figure 1G-I), and BSP was located in the osteoblast cytoplasm (Figure 1J-L). All the markers showed the same profiles, with increased expression at 21 days of differentiation.

### Spheroid morphology and growth curves

**Figure 2:**
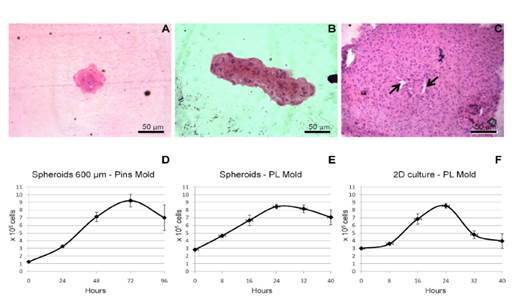
Spheroid morphology and growth. Spheroids were grown in three different microenvironments and then processed for histological examination. **(A)** it is possible to observe a typical type "A" spheroid grown for 24 hours in a 600 µm agarose mold. This type of spheroid assumed spherical morphology with well-grouped cells. **(B)** it is possible to observe a typical type "B" spheroid grown in a 600 µm agarose mold for 24 hours and then transferred to a periapical lesion mold. This type of spheroid presented a compact and spindle-shaped morphology. **(C)** it is possible to observe a typical type "C" spheroid, which was grown from cells from 2D monolayer culture transferred to a periapical lesion mold. The type "C" spheroid thus grown showed a spherical morphology comparable to the type "A" spheroids but larger and with a hollow nucleus without cells (arrows). **(D-F)** The three types of spheroid's growth curves presented the growth, stationary, and decline phases. Time-point values are presented as mean and ± s.d.

Spheroid morphology was determined by optical microscopy of histological sections stained with H&E. Figure 2 shows histological sections of three types of spheroids, which were formed with different cell inocula and agarose molds.

The 3D 600-µm spheroids, i.e., those formed during 24 hours after inoculation of SHED into the 600-µm agarose pin molds, presented a spherical morphology, with well- grouped cells forming small and compact spheroids (Figure 2A).

The 3D-PL spheroids, i.e., those that were formed early during 24 hours in the 600-µm agarose pin molds and then transferred to the periapical lesion molds, presented a compact and spindle-shaped morphology (Figure 2B).

The 2D-PL spheroids from SHED cultured in monolayers and then transferred to the periapical lesion molds showed a spherical morphology comparable to that of the 3D 600-µm spheroids but had larger sizes. In this type of spheroid, we observed a hollow nucleus without cells (Figure 2C).

Three spheroid growth curves were constructed according to the initial cell inoculum and the type of agarose mold (600-µm pin mold or periapical lesion mold). In all three types of curves, the growth, stationary, and decline phases were observed, and the last phase presented proportionally more dead cells than living cells.

The growth of the 3D 600-µm spheroids, i.e., those formed when SHED from monolayer cultures were seeded in 600-µm molds, occurred between 24 and 48 hours (Figure 2D). The growth of the 3D-PL spheroids, i.e., those produced when early 24-hour spheroids formed in 600-µm molds were transferred to periapical lesion molds, occurred immediately from the time of inoculation until 24 hours after inoculation (Figure 2E). The growth of the 2D-PL spheroids, i.e., those formed when SHED was seeded in periapical lesion molds, occurred between 8 and 16 hours (Figure 2F).

### Viability of SHED spheroids

**Figure 3:**
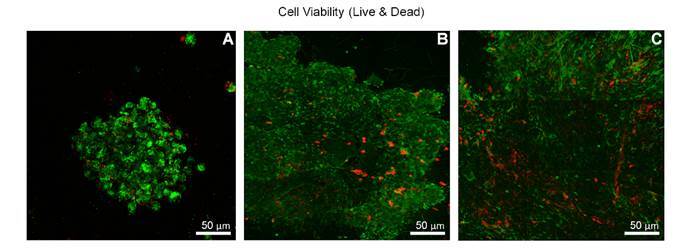
Viability of SHED grown in spheroids. For spheroids, viability was assessed using fluorescence microscopy (Live / Dead kit). **(A)** Type "A" spheroids, grown for 24 hours (600 µm mold), showed a high rate of live cells (stained green) with some dead cells (stained red). **(B)** Type "B" spheroids, grown for 24 hours in 600 µm mold and then transferred to periapical lesion mold, are spindle-shaped and have many alive and some dead cells. **(C)** Type "C" spheroids, SHED grown in monolayer and then transferred to periapical lesion mold, also showed most living cells, but with the central region composed of dead cells.

The viability of the 3D 600-µm spheroids collected after 24 hours of development was high, as detected by the live/dead test with fluorescence microscopy. Spheroids exhibited a spherical morphology, with many live cells stained green throughout their length and fewer apoptotic cells stained red (Figure 3A).

The 3D-PL spheroids were predominantly fusiform, with most cells viable and only a few apoptotic cells dispersed on the periphery (Figure 3B).

The 2D-PL spheroids showed a spheroidal morphology, with most living cells on the periphery and a cluster of apoptotic cells in the central region, which is typical of multicellular spheroids (Figure 3C).

### Transcriptional expression of mesenchymal stemness and osteoblastic differentiation markers

**Figure 4:**
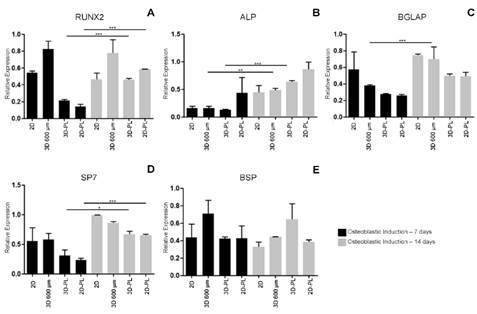
Relative expression of the transcripts that encode osteoblastic differentiation markers (RUNX2, ALP, BGLAP, SP7 and BSP) in the samples: SHED grown in 2D monolayer culture (2D), spheroids grown in 600 μm molds (3D 600 µm), spheroids grown in 600 µm and transferred to periapical lesion molds (3D-PL) and SHED grown in 2D monolayer culture and transferred to periapical lesion molds (2D-PL) for 7 and 14 days. GAPDH was used as a housekeeping expression. The difference between the groups was assessed using the *t* statistical test. *** p <0.001; ** p <0.01; * p < 0.05. The appropriate independent triplicates were performed.

The relative transcription levels of the osteoblastic differentiation markers RUNX2, ALP, BGLAP, SP7 and BSP in SHED cultured in 2D monolayers or in 3D molds for spheroid formation during osteoblastic differentiation for 7 to 14 days were evaluated by RT‒qPCR. The RUNX2 mRNA was significantly differentially expressed in both types of samples (SHED grown in 2D monolayers or in 3D spheroids formed in periapical lesion molds) (Figure 4A). The ALP mRNA was differentially expressed when 3D spheroids were formed either in the 600-µm or in the periapical lesion molds (Figure 4B). The BGLAP mRNA was differentially expressed in 3D spheroids formed in the 600- µm molds (Figure 4C). SP7 was equally differentially expressed in both SHED cultured in 2D monolayers and in 3D spheroids formed in the molds of the periapical lesion (Figure 4D). Although BSP mRNA was expressed, its profiles did not differ between the tested samples (Figure 4E).

**Supplemental Figure 3:**
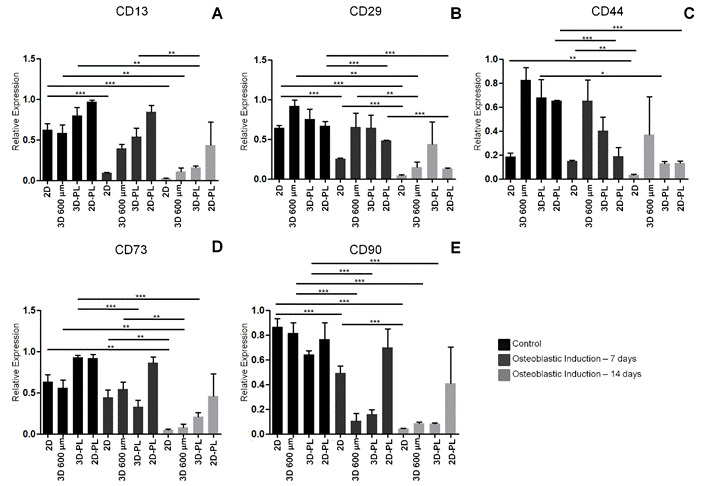
Relative expression of the transcripts that encode stemness markers (CD13, CD29, CD44, CD73 and CD90) in the samples: SHED grown in 2D monolayer culture (2D), spheroids grown in 600 μm molds (3D 600 µm), spheroids grown in 600 µm and transferred to periapical lesion molds (3D-PL) and SHED grown in 2D monolayer culture and transferred to periapical lesion molds (2D-PL) comparing undifferentiated (control) with osteoblastic differentiated cells for 7 and 14 days. GAPDH was used as a housekeeping expression. The difference between the groups was assessed using the ANOVA One-Way test. *** p <0.001; ** p <0.01; * p <0.05. The appropriate independent triplicates were performed

The mRNA levels of the mesenchymal stemness markers CD13, CD29, CD44, CD73, and CD90 were also tested. The transcription of these markers in different samples (SHED cultured in 2D monolayers and spheroids formed either in 600-µm or in periapical lesion molds) significantly decreased during osteoblastic differentiation (Supp. Fig. 3A- E).

## Discussion

The persistence, recurrence, and even refractoriness of periapical lesions in patients to synthetic materials used in endodontic treatment represent important difficulties for regenerative endodontics, including the rate of success in periapical lesion healing. Hence, we investigated whether spheroids originating from SHED could be used in the biological restoration of such lesions, as previously considered ^20^.

The causes of these complications may be due to the complexity of the root canal system, leakage of the filling materials, extra root infections, and actual cystic lesions ^21^. The intrinsic factors of the host must also be considered, such as the immune response in periapical lesion repair ^4^.

Genetic polymorphisms have been associated with many endodontic phenotypes. An imbalance in the expression of genes involved in the healing process may influence the outcome of endodontic treatment, such as the resistance or recurrence of periapical lesions ^4^ ^,^ ^22^.

The aim of this study was to establish an *in vitro* model system to test the biological restoration of a periapical lesion cavity using SHED. The growth behaviors of SHED were compared in monolayers (2D) and spheroids (3D) using an artificial model, which was assembled by 3D printing and mimicked a periapical lesion of an upper lateral incisor tooth.

SHED is promising for use in bone regeneration because of their low immunogenicity, high proliferation rate, and responsiveness to differentiation inducers. Additionally, they are more easily accessible than dental pulp stem cells (DPSCs) from permanent teeth ^6^ ^,^ ^12^.

A tomography image of a periapical lesion of an upper lateral incisor tooth was used to make a negative impression of the lesion in acrylic using a 3D printer. From the negative mold, a positive mold in agarose was prepared and filled with SHED from monolayer culture or spheroids.

During the formation of tissues *in vivo*, cells interact with each other through extracellular matrix components and assume a 3D organization ^23^. We used spheroids to fill the periapical lesion mold, with subsequent differentiation of SHED into osteoblasts.

Then, the osteoblastic differentiation of SHED and spheroids was tested. SHED from monolayers proliferated and showed increased viability.

The activity of the alkaline phosphatase (ALP) protein increased in the initial periods and decreased in the later stages of differentiation. This finding was consistent with literature data since ALP represents a marker of the early stages of osteoblastic differentiation ^24^. The mineralization process and the immunolocalization of the RUNX2, ALP, and BSP protein markers were prominent in the late phase of differentiation. These findings showed that the SHED-based *in vitro* differentiation model system was adequate.

SHED spheroids presented a spherical morphology and had a high viability. The growth curves demonstrated exponential, stationary, and decline phases, as expected.

After being inoculated in the periapical lesion mold, in less than 24 hours, spheroids started to aggregate together, finally generating larger spheroids with different shapes and high viability. This property might be important when using spheroids in the endodontic treatment of periapical lesions.

SHED cultured in a 2D monolayer and directly inoculated into the periapical lesion mold generated single large spheroids, which were formed in less than eight hours. In these spheroids, viable cells were mainly located on the surface, and apoptotic cells were in the central zone.

The location of viable cells is a characteristic trait of large spheroids, which can form a hollow vascular lumen-like center through the apoptosis of polarized central cells, forming a layer of dead cells resulting from hypoxia due to the difficulty of oxygen delivery. The nutrition of cells located in the central spheroid area is also hampered ^25^, which may have caused a decrease in cell viability compared with that of preformed smaller spheroids, as both types were inoculated into the periapical lesion mold.

Analysis of the transcription of the respective mRNAs encoding the mesenchymal stem cell markers (stemness markers) CD13, CD12, CD44, CD73 and CD90 revealed that the expression of these genes decreased over time as osteoblastic differentiation progressed. Concomitantly, the mRNA expression of the differentiation markers RUNX2, ALPL, BGLAP, and SP7 increased at the beginning of osteoblastic differentiation (7 to 14 days) in both monolayer and spheroid SHED cultures, while BSP mRNA expression remained uniform during this period.

Biological restoration of the periapical lesion cavity represents an attractive option for use in endodontics. It may be possible to establish a clinical protocol that begins with SHED spheroid implantation at the beginning of osteoblastic differentiation. Over time and with the occurrence of mineralization, the lesion and the cells would be gradually replaced by *in situ* formed bone tissue, which would rule out any adverse reactions from the host.

There are limitations to this study that may impact the generalizability of the results to broader *in vivo* and population contexts. First, considering the *in vitro* nature of the study, the obtained results may not fully reflect the cell behavior in the *in vivo* environment. Controlled culture conditions do not accurately represent the complexity of biological systems in the human body. Additionally, using a primary cell line may pose challenges owing to difficulty in obtaining these cells because of their human origin. However, the existence of already characterized SHED lines could meet this need. Moreover, it will be necessary to consider any differences in treatment success among patients, as they will have different genetic backgrounds.

In this study, we developed an *in vitro* model system that mimics a periapical lesion with the possibility of repopulating the lesion cavity with 3D spheroids of SHED.

## Conclusion

Among the types of spheroids that we constructed, the one that showed the best results was type 3D-PL; that is, the spheroids started inside a 600-µm mold and then continued to develop inside the periapical lesion mold. Additionally, the model allowed the differentiation of spheroid cells into osteoblasts that mineralized over time. This opens a new perspective for endodontics by restoring areas with periapical lesions through the replacement with *in situ* formed bone tissue.
